# The left main bronchus transected incorrectly during video-assisted thoracoscopic lobectomy: a case report

**DOI:** 10.1186/s40792-020-01073-8

**Published:** 2020-11-19

**Authors:** Yuma Shindo, Masahiro Miyajima, Yasuyuki Nakamura, Wataru Arai, Ryunosuke Maki, Kodai Tsuruta, Atsushi Watanabe

**Affiliations:** Department of Thoracic Surgery, Sapporo Medical University School of Medicine, South-1, West-16, Chuo-ku, Sapporo, 060-8543 Japan

**Keywords:** Intraoperative complication, Incorrect transection, Main bronchus, Sleeve bronchoplasty

## Abstract

**Background:**

Several severe intraoperative complications of lung cancer surgery have been reported, but the incorrect transection of the main bronchus is a very rare and serious complication. We report a surgical case of a patient with left lower lobe lung cancer invading the inferior segment of the lingula, with fused interlobar fissure and dense pleural adhesion, in which the left main bronchus was mistaken for the left lower lobe bronchus and was transected.

**Case presentation:**

A 64-year-old woman with lung adenocarcinoma was referred to our hospital for surgical treatment. Chest computed tomography (CT) scan showed a 30-mm nodule with a clear border and irregular margins in the center of the anterior (S8) segment of the lower lobe of the left lung and another similar 30-mm nodule in the lateral (S9) segment of the same lobe. Metastasis within the same lobe was suspected. A thoracoscopic left lower lobectomy was scheduled for the patient. As the patient had a moderately, fused fissure, dense pleural adhesion, and suspicious tumor invasion from the left S8 segment to the left S5 segment, and the interlobar node tightly adhered to the main PA at the site of basilar artery origin of the LLL, we performed left lower lobectomy and a left S5 segmentectomy using the fissureless fissure-last technique. During surgery, the left main bronchus was mistaken for the left lower lobe bronchus and was transected. After transecting the left main bronchus, we performed a sleeve bronchoplasty to prevent pneumonectomy.

**Conclusions:**

We experienced the rare and serious intraoperative complication of the incorrect transection of the main bronchus. There are few reports of this intraoperative complication, and it should not be overlooked by surgeons.

## Background

Thoracotomies, robot-assisted surgeries, and thoracoscopic approaches are all used in the surgical treatment of lung cancer and severe intraoperative complications are possible in any approach. Many incorrect transections of bronchovascular structures have been reported in previous reports, but it is rare that the main bronchus is mistaken for the lobe bronchus and transected. We report the case of a patient who underwent lower left lobectomy and left S5 segmentectomy for lung cancer in the lower lobe of the left lung that invaded the inferior segment of the lingula. During the surgery, the left main bronchus was mistaken for left lower lobe bronchus and transected. A successful sleeve bronchoplasty was then performed to avoid a pneumonectomy.

## Case presentation

**Fig. 1:**
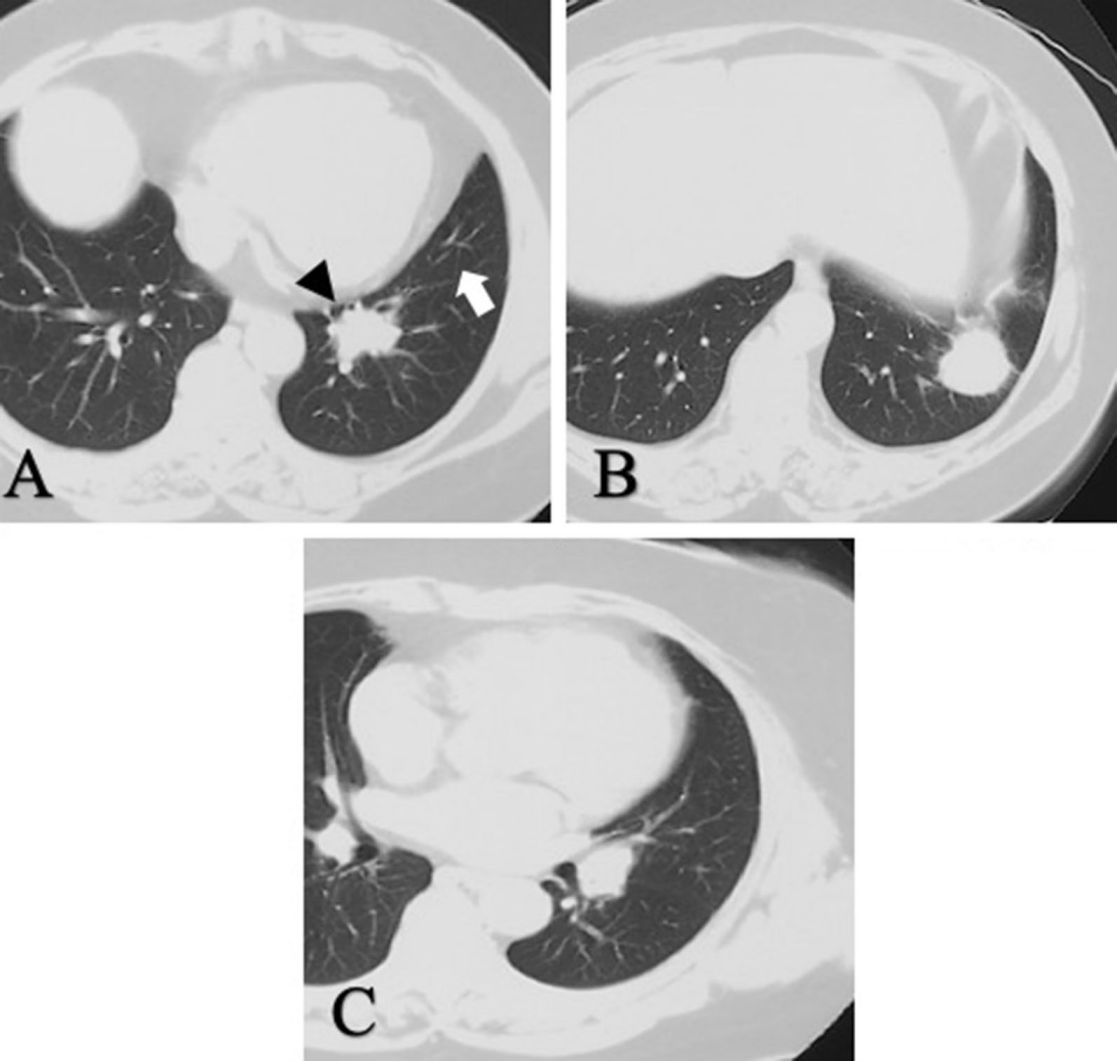
Computed tomography scan results showing two pulmonary nodules and swollen interlobar node. **a** A 30-mm nodule in the center of the antero-basal segment of the left lower lobe. Suspicious interlobar invasion of S8 tumor (black arrowhead) and interlobar fissure (white arrow). **b** Another nodule in the lateral segment of the left lower lobe. **c** A swollen interlobar lymph node

**Fig. 2:**
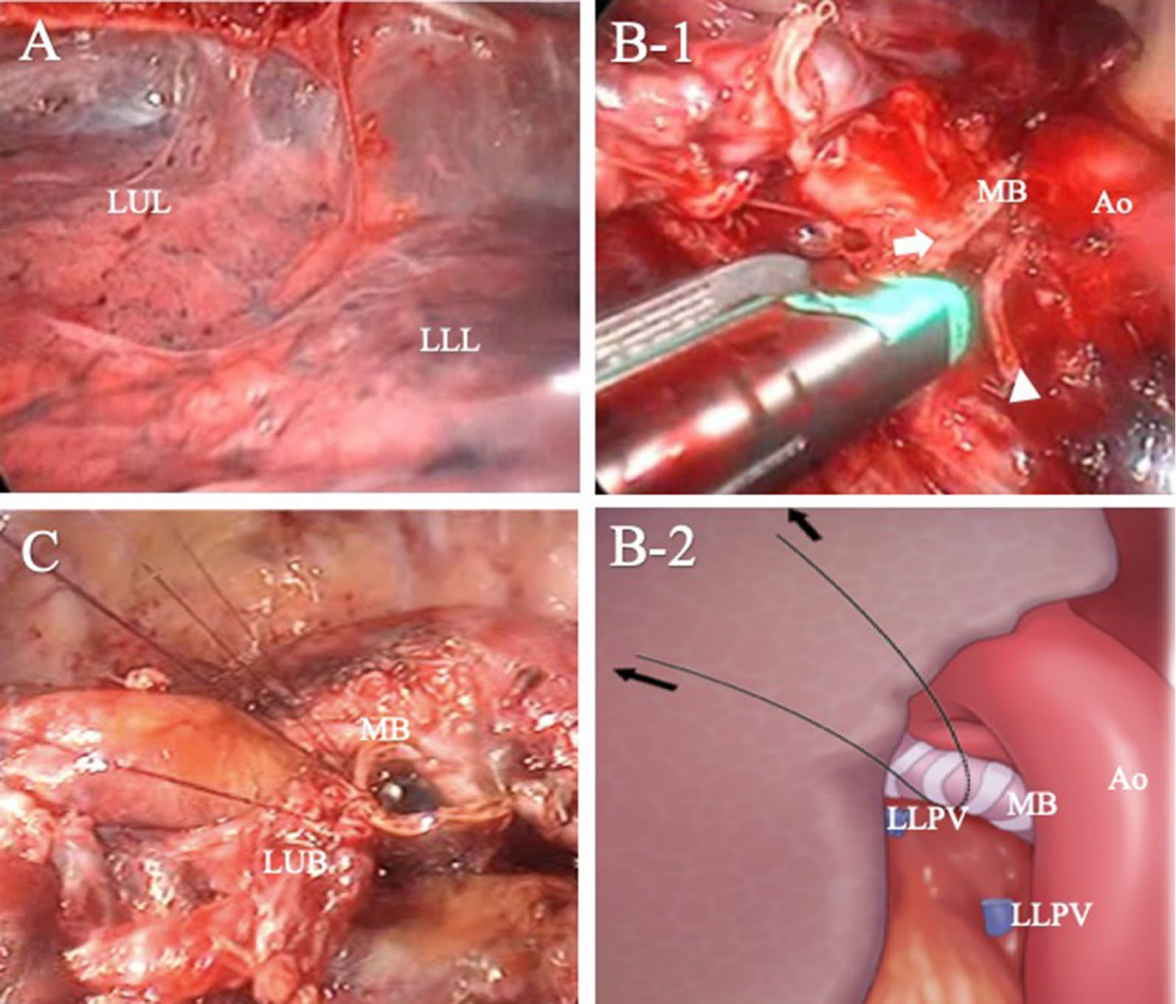
The intraoperative views. **a** A fused fissure was present between the upper lobe and the left lower lobe. **b-1** The main bronchus was mistaken for the left lower lobe bronchus and erroneously transected. Proximal stump (arrowhead), distal stump (arrow). **b-2** The schema of **b-1**. **c** Sleeve bronchoplasty was performed with the anastomosis of the left main bronchus and the left upper lobe bronchus

A 64-year-old woman with no significant medical history presented with hemosputum and underwent examinations at a referral hospital. Her physical findings, tumor markers, and other laboratory tests were unremarkable. A spirometry test revealed obstructive ventilatory disturbance. A chest computed tomography (CT) scan showed a 30-mm nodule with a clear border and irregular margins in the center of the S8 segment of the lower lobe of the left lung and another similar 30-mm nodule in the S9 segment of the same lobe with a swollen interlobar lymph node. Pulmonary metastasis within the same lobe was suspected (Fig. 1). After a transbronchial lung biopsy was done, the patient was diagnosed with cT3N1M0 Stage IIIA lung adenocarcinoma in accordance with the 8th edition of the TNM classification system from the Union for International Cancer Control, and she was referred to our hospital for surgery. During preoperative examinations including bronchoscopy or chest CT, there was no findings the anatomical variations of the left airway. A thoracoscopic left lower lobectomy was scheduled for the patient. Under general anesthesia with epidural block and one-lung ventilation, a 40-mm thoracotomy in the fifth intercostal space on the midaxillary line was performed, and two trocar ports were placed: one in the seventh intercostal space on the anterior axillary line and the other in the eighth intercostal space on the posterior axillary line. The left thoracic cavity was observed via thoracoscopy. As a moderate, fused fissure was found (Fig. 2a) and the S8 tumor suspiciously invaded to S5 of the lingula in ventral interlobar fissure, we did not perform interlobar division. Therefore, we performed a left lower lobectomy using the fissureless fissure-last technique, and a combined left S5 segmentectomy. First, the pulmonary ligament was transected and lymph node (#9) was dissected. The left lower pulmonary vein was exposed from the hilar region. It was taped and transected with an endoscopic stapler. Next, the bronchus was transected while the lower lobe was pulled to the superior ventral side. The left lower lobe bronchus was taped and transected with an endoscopic stapler (Fig. 2b). The superior pulmonary artery (A6) was exposed and dissected. As the basilar pulmonary artery adhered tightly to the bronchial lymph nodes, the left main pulmonary artery trunk was taped at the just proximal site of the A6 and the pulmonary artery was clamped at this level. Then, the basilar pulmonary artery of the lower lobe was excised including the A5 origin, and the arterial stump was closed with 4-0 Prolene running suture. The A4 was bluntly oppressed at the distal side to prevent backflow of blood during the procedure. After the pulmonary artery closure, we noticed that we had erroneously transected the left main bronchus. We converted to a thoracotomy and tried to perform a left upper sleeve lobectomy with an inferior segmentectomy of the lingula. Therefore, we transected inferior bronchus (B5) of the lingula at the origin with an endoscopic stapler and the inferior pulmonary vein of the lingula was doubly ligated and transected. The intersegmental was divided along the caudal margin of inferior vein of the lingual (V4) with endoscopic staplers. Finally, sleeve bronchoplasty was performed with the anastomosis of the left main bronchus and the left upper lobe bronchus with 4-0 Prolene running suture (Fig. 2c). The pericardial fat pedicle was harvested and used to cover the bronchial anastomosis. After removing the lower left lobe and S5 of the lingula, a lymph node dissection was performed to complete the operation. The operation time was 5 h and 18 min, and the estimated blood loss was 360 ml.

Histopathology revealed that the tumor was an adenocarcinoma with peribronchial lymph node involvement (T4N1M0 stage III A). The patient’s postoperative course was uneventful until her death from primary cholangiocarcinoma 3 years after the lung resection.

## Discussion

Incorrect transection of the main bronchus is a severe intraoperative complication of lung cancer surgery, and has been reported to occur in 0.9–1.8% of thoracoscopic surgery pulmonary resections performed by surgeons at various levels of experience [1, 2]. Most of these types of complications are the incorrect transection of the bronchus intermedius and lobe bronchus, and few cases involve the main bronchus. Four cases of the incorrect transection of the main bronchus have been reported (Table 1) [3–6].

**Table 1:** The cases of the incorrect transection of the main bronchus

Patient age, sex	Authors/published year	Side	Scheduled surgery	Treatment	Fissureless technique
61, M	Flores RM/2011 [3]	Left	LUL	Pneumonectomy	Yes
Unknown	Liang C/2013 [4]	Right	RLL	Bronchoplasty	Unknown
Unknown	Reis JE/2017 [5]	Left	Unknown	Bronchoplasty	Unknown
Unknown	Amer K/2011 [6]	Left	LUL	Bronchoplasty	Yes
Present	Shindo	Left	LLL	Bronchoplasty	Yes

All additional treatments were performed after conversion to thoracotomy

*LUL* left upper lobectomy, *RLL* right lower lobectomy

According to these reports, the fissureless technique may lead to incorrect transections. The surgeon must be aware of all anatomical structures inside the chest when using the fissureless technique [1, 6]. In this case, we attempted to cut the bronchus while pulling the lower lobe to the superior ventral side. However, as an interlobar fissure was not created, we misidentified the positional relationship between the lung and the bronchi. In addition, we routinely perform a ventilation test before transecting the bronchus, whereas, in this case, the ventilation of the left upper lobe during the test was not adequately observed because of the superior ventral side oppression of the left lower lobe. For prevention from this anatomical misunderstanding when using the fissureless technique, we must recognize that the position of the left main bronchus is significantly shifted due to the strong traction of the left lower lobe to the superior ventral side. Furthermore, we need to confirm the origin of left upper bronchus or superior segmental bronchus (B6) by peeling between the left main pulmonary artery and the main bronchus from dorsal side. In this case, the biggest problem is that we transected the bronchus with an endoscopic stapler, even though we could not confirm the insufflation of the remnant of the left lungs. If it is not confirmed, we should have released the superior ventral traction and returned to the normal position or used bronchoscope for confirmation.

Pneumonectomy or bronchoplasty can be considered treatments for the incorrect transection of the main bronchus. Table 1 shows that all cases were transferred to thoracotomy and bronchoplasty was performed in most of the reported cases. Our patient underwent a sleeve bronchoplasty.

## Conclusion

The incorrect transection of the main bronchus is a rare complication of lung surgery, however, it can occur at all levels of surgical experience. This complication should not be overlooked and bronchoplasty should be considered in order to avoid a pneumonectomy when it occurred.

## Data Availability

Date sharing is not applicable to this article as no datasets were generated or analyzed for the study.

## Abbreviations

LUL: Left upper lobectomy
LLL: Left lower lobectomy
RLL: Right lower lobectomy
MB: Main bronchus
LUB: Left upper bronchus
Ao: Aorta
LLPV: Left lower pulmonary vein
